# Supplementary material for: Proton magnetic resonance spectroscopy in frontotemporal lobar degeneration-related syndromes
Source: Neurobiol Aging. Author manuscript; Available in PMC 2022 Mar 1. (PMC8776136; doi:10.1016/j.neurobiolaging.2021.10.012)
Supplement: Supplementary material [file EMS140676-supplement-Supplementary_material.docx]

# Supplementary materials

## Figure S1: MRS Voxel position.


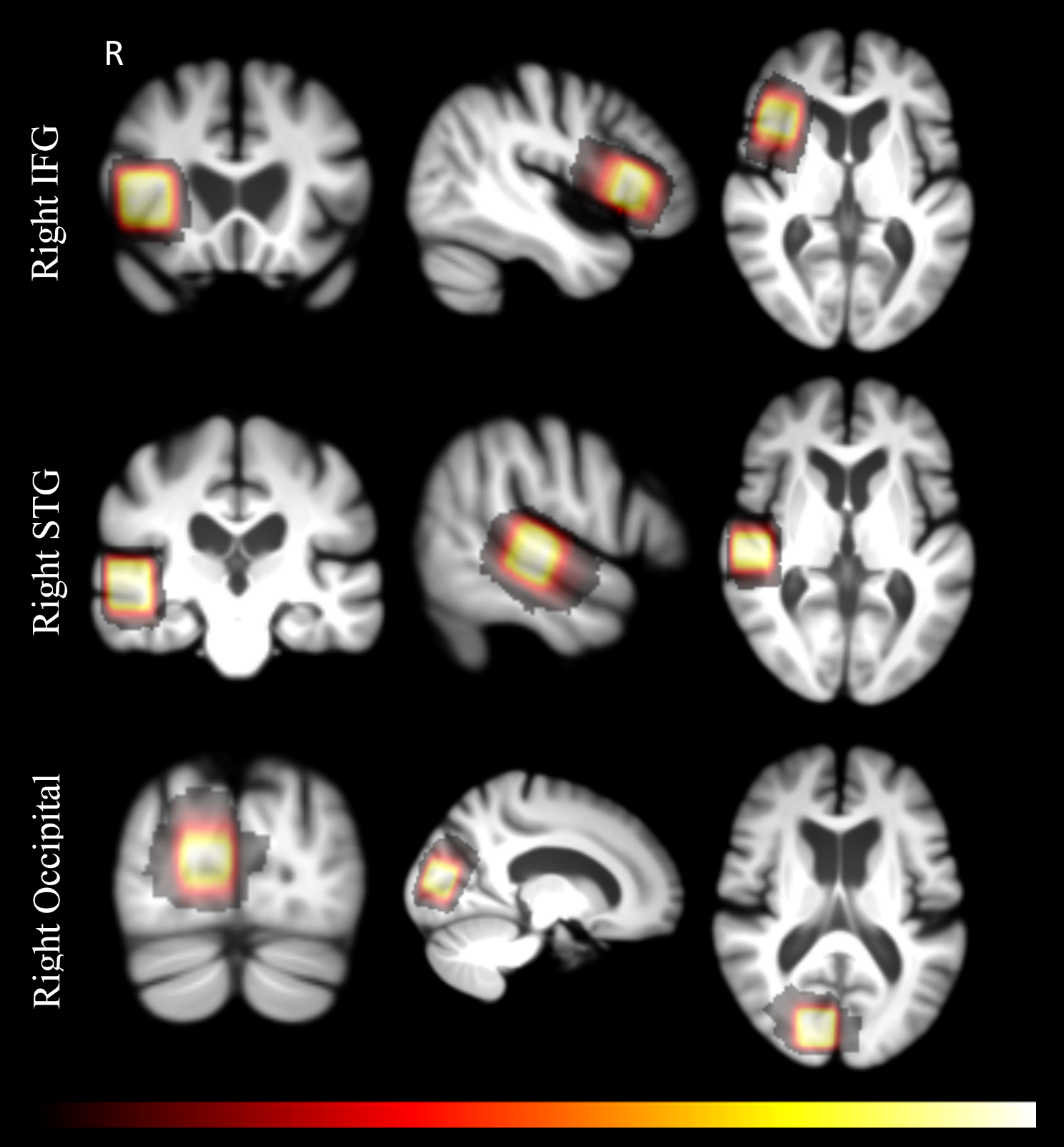


Map of all participants’ MRS regions of interest superimposed on a mean structural image. IFG: inferior frontal gyrus. STG: superior temporal gyrus. Colour bar ranges from 0 to all participants (98).

## Table S1: MRS Quality Metrics

|  | **Mean (SD)** | **Control** | **FTLD** | **t** | **df** | **p** |
| --- | --- | --- | --- | --- | --- | --- |
| **Right IFG** | **Glutamate CRLB** | 3.92 (0.43) | 4.75 (1.64) | -3.72 | 71.00 | <0.001 |
|  | **NAA+NAAG CRLB** | 1.95 (0.23) | 2.07 (0.45) | -1.75 | 92.38 | 0.08 |
|  | **LW** | 7.33 (0.79) | 6.32 (1.16) | 5.13 | 95.50 | <0.001 |
|  | **SNR** | 56.40 (5.19) | 47.28 (8.62) | 6.53 | 95.82 | <0.001 |
| **Right STG** | **Glutamate CRLB** | 4.25 (1.03) | 4.90 (1.37) | -2.47 | 82.00 | 0.02 |
|  | **NAA+NAAG CRLB** | 1.94 (0.33) | 2.104 (0.59) | -1.57 | 76.72 | 0.12 |
|  | **LW** | 8.95 (0.90) | 8.41 (1.56) | 1.99 | 77.42 | 0.05 |
|  | **SNR** | 49.69 (9.34) | 45.29 (11.29) | 1.95 | 81.16 | 0.05 |
| **Right OCC** | **Glutamate CRLB** | 5.10 (2.90) | 4.69 (2.80) | 0.64 | 59.53 | 0.53 |
|  | **NAA+NAAG CRLB** | 1.87 (0.81) | 1.76 (0.72) | 0.62 | 56.64 | 0.54 |
|  | **LW** | 7.48 (0.81) | 7.22 (0.82) | 1.38 | 63.15 | 0.17 |
|  | **SNR** | 58.13 (16.00) | 61.55 (13.81) | -1.00 | 55.08 | 0.32 |

Magnetic resonance spectroscopy quality metrics. IFG: inferior frontal gyrus. STG: superior temporal gyrus. OCC: Occipital lobe. FTLD: Frontotemporal lobar degeneration. CRLB: Cramér-Rao Lower Bound. LW: water linewidth. SNR: Signal to noise ratio.

## Table S2: Cognitive test results including subscores.

|  | Control | FTLD  (all groups) | bvFTD | PSP | CBS | PPA | t | df | p |
| --- | --- | --- | --- | --- | --- | --- | --- | --- | --- |
| Addenbrookes Cognitive Examination – Revised subscores | | | | | | | | | |
| Atten/orient | 18  (0) | 15.45  (3.90) | 15.5 (3.4) | 16 (3.3) | 15.8 (2.8) | 13.8 (6) | NA | NA | NA |
| Memory | 24.6 (1.4) | 18.67  (7.09) | 17.2 (6.2) | 20.4 (5.5) | 21.2 (6.4) | 13.9 (9.3) | 6.3 | 66.6 | <0.001 |
| Fluency | 12.7 (1) | 6.45  (3.7) | 5.8 (3.8) | 6.3 (3.2) | 7.9  (4) | 6.1 (4.5) | 12.3 | 72.6 | <0.001 |
| Language | 25.5 (0.5) | 21.22  (5.78) | 20.1 (6.1) | 23.8 (2.1) | 22.1 (3.9) | 15.8 (8.5) | 5.7 | 60.5 | <0.001 |
| Visuospatial | 15.7 (0.4) | 12.20  (4.12) | 13.7 (2.8) | 11.7 (4.2) | 11.5 (3.8) | 12.5 (5.2) | 6.4 | 61.2 | <0.001 |
| Total | 96.5 (2.4) | 73.98 (20.53) | 72.4 (18.3) | 78.2 (14.9) | 78.5 (18.7) | 62.1 (30.2) | 8.4 | 61.5 | <0.001 |
| FAB | 17.1 (0.8) | 11.66  (4.52) | 10.7 (5.4) | 12.1 (3.8) | 11.3 (5.7) | 12 (4.4) | 9.1 | 64.3 | <0.001 |
| Cambridge Behavioural Inventory - Revised subscores | | | | | | | | | |
| Memory | 0.1 (0.3) | 9.67  (7.12) | 16.1 (5.5) | 8.1 (6.2) | 6.1 (4.6) | 10.6 (8.7) | -10.4 | 59.3 | <0.001 |
| Everyday skills | 0 (0) | 9.19  (6.38) | 9.1 (5.7) | 10.5 (6.8) | 8.5 (6.9) | 7.2 (5.7) | NA | NA | NA |
| Self care | 1.3 (1.3) | 5.04  (4.91) | 3 (3.4) | 7 (5.3) | 6.3 (4.8) | 1.6 (2.5) | -5.7 | 71.8 | <0.001 |
| Abnormal behaviour | 0.9 (1.4) | 5.47  (5.86) | 13.1 (6.1) | 4.2 (4.8) | 3.8 (4.3) | 2.7 (2.7) | -5.8 | 69.2 | <0.001 |
| Mood | 0 (0) | 3.87  (3.34) | 5.9 (3.2) | 3.4 (2.8) | 5.4 (4.3) | 1.7 (2) | NA | NA | NA |
| Beliefs | 0.4 (1) | 0.74  (1.96) | 1.9 (3.7) | 0.4 (1.1) | 0.7 (1.6) | 0.5 (1.2) | -1.2 | 92.1 | 0.2 |
| Eating | 1.1 (1.1) | 4.50  (4.65) | 10.2 (5) | 3.9 (3.9) | 2.2 (3.2) | 2.7 (2.7) | -5.4 | 68.6 | <0.001 |
| Sleep | 1 (1.3) | 3.55  (2.36) | 4 (2) | 3.6 (2.3) | 4.6 (2.3) | 2.1 (2.4) | -6.9 | 94.1 | <0.001 |
| Motor behaviour | 0.8 (1.5) | 4.91  (5.15) | 12.5 (2.5) | 3.3 (3.8) | 3.4 (4.1) | 2.8 (4.2) | -5.7 | 74.4 | <0.001 |
| Motivation | 0.6 (1) | 7.72  (5.98) | 13.5 (4.3) | 7.7 (5.9) | 6 (4.8) | 4 (4.9) | -9.0 | 64.5 | <0.001 |
| Total | 6.2 (6.1) | 54.65 (32.97) | 89.3 (22.7) | 52 (31.6) | 46.9 (27.8) | 35.8 (27) | -11.1 | 65.2 | <0.001 |
| Clinician Rating (score based on presence/absence of features) | | | | | | | | | |
| Behaviour | 0 (0) | 3.6  (3.37) | 8.9 (1.3) | 3.3 (2.7) | 1.5 (1.4) | 1.4 (1.7) | NA | NA | NA |
| Visuospatial | 0 (0) | 1.15  (1.34) | 0.1 (0.3) | 0.6 (0.8) | 3.1 (0.9) | 1.6 (1.2) | NA | NA | NA |
| Language | 0 (0) | 2.52  (2.28) | 2.6 (2.6) | 1.5 (1.5) | 2.4 (1.6) | 4.8 (2.4) | NA | NA | NA |

Cognitive profile of the study participants. FTLD: frontotemporal lobar degeneration. bvFTD: behavioural variant frontotemporal dementia. PSP: progressive supranuclear palsy. CBS: corticobasal syndrome. PPA: primary progressive aphasia (all subtypes). FAB: frontal assessment battery.

## Figure S1: Brain volume within the MRS region of interest


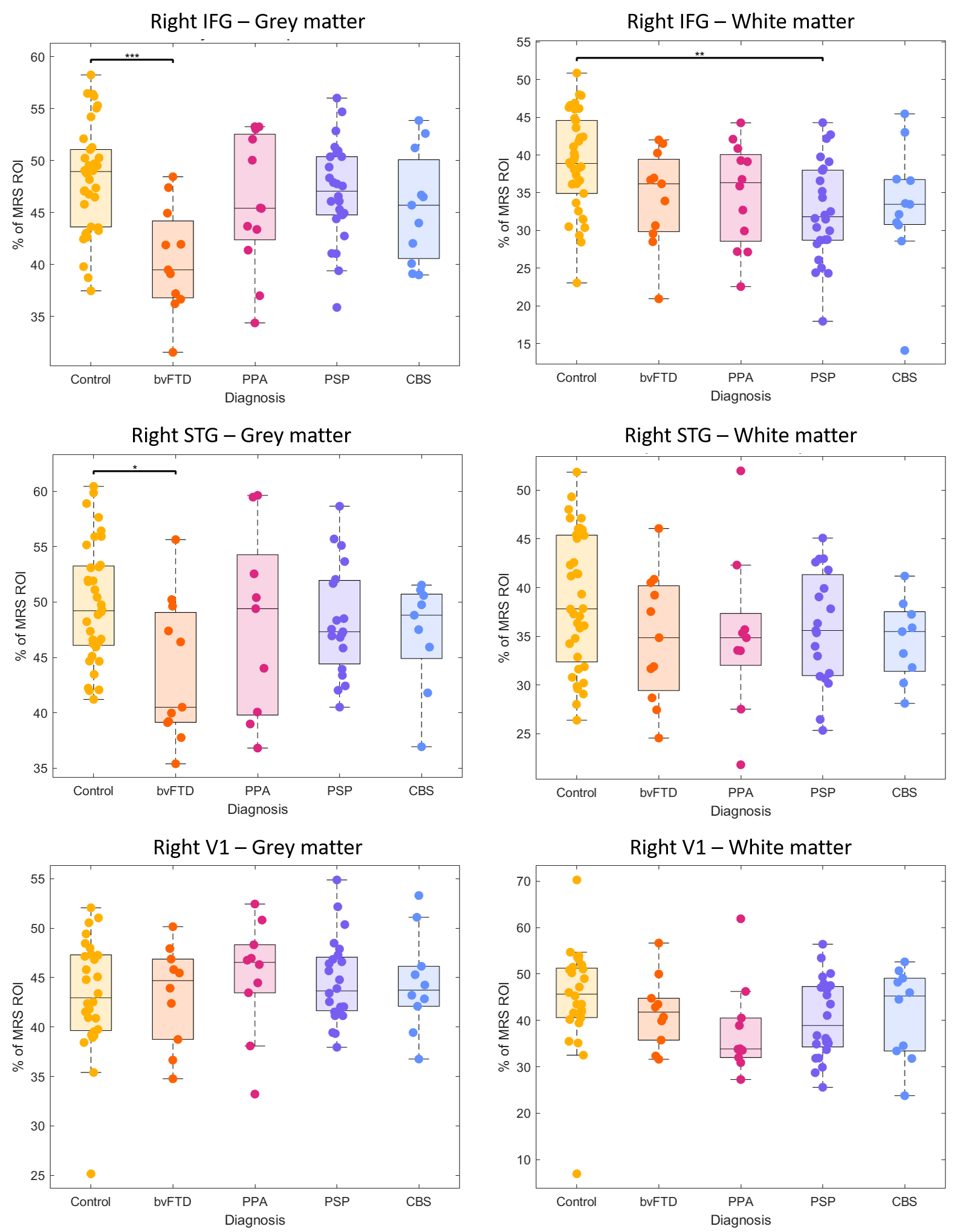


MRS: Magnetic resonance spectroscopy. ROI: region of interest/voxel. FTLD: Frontotemporal lobar degeneration. NAA: N-acetyl-aspartate. NAAG: N-acetyl-aspartyl-glutamate. IFG: Inferior frontal gyrus. STG: Superior temporal gyrus. V1: Primary visual cortex/occipital lobe. bvFTD: behavioural variant frontotemporal dementia. PSP: progressive supranuclear palsy. CBS: corticobasal syndrome. PPA: primary progressive aphasia (all subtypes).

## Table S3: ANOVA of partial volume corrected MRS results

| **ANOVA (between and within subject effects)** | | | | | | |
| --- | --- | --- | --- | --- | --- | --- |
| **Cases** | | **Sum of Squares** | **df** | **Mean Square** | **F** | **p** |
| ROI | | 4.05 | 2 | 2.03 | 4.52 | 0.013 |
| ROI x Diagnosis | | 4.40 | 8 | 0.55 | 1.23 | 0.288 |
| Metabolite | | 10280.33 | 8 | 1285.04 | 2841.54 | <0.001 |
| Metabolite x Diagnosis | | 31.33 | 32 | 0.98 | 2.17 | <0.001 |
| ROI ✻ Metabolite | | 149.26 | 16 | 9.33 | 55.58 | <0.001 |
| ROI x Metabolite x Diagnosis | | 18.54 | 64 | 0.29 | 1.73 | <0.001 |
| Diagnosis | | 5.29 | 4 | 1.32 | 0.91 | 0.464 |
| **Simple Main Effects - Diagnosis** | | | | | | |
| **Level of ROI** | **Level of Metabolite** | **Sum of Squares** | **df** | **Mean Square** | **F** | **p** |
| Frontal | Aspartate | 1.19 | 4 | 0.30 | 1.87 | 0.125 |
|  | Glutamine | 0.80 | 4 | 0.20 | 1.05 | 0.391 |
|  | **Glutamate** | **7.67** | **4** | **1.92** | **5.73** | **<0.001** |
|  | Myoinositol | 2.94 | 4 | 0.73 | 1.41 | 0.240 |
|  | Asc/GSH | 0.43 | 4 | 0.11 | 1.41 | 0.239 |
|  | Glucose/Tau | 3.87 | 4 | 0.97 | 2.34 | 0.064 |
|  | Choline | 0.10 | 4 | 0.02 | 0.61 | 0.654 |
|  | **NAA** | **8.83** | **4** | **2.21** | **4.16** | **0.004** |
|  | Creatine | 0.41 | 4 | 0.10 | 0.63 | 0.646 |
| Temporal | Aspartate | 0.58 | 4 | 0.14 | 0.14 | 0.965 |
|  | Glutamine | 1.19 | 4 | 0.30 | 1.02 | 0.404 |
|  | **Glutamate** | **7.26** | **4** | **1.82** | **2.71** | **0.037** |
|  | Myoinositol | 1.17 | 4 | 0.29 | 0.85 | 0.499 |
|  | Asc/GSH | 0.40 | 4 | 0.10 | 0.45 | 0.776 |
|  | Glucose/Tau | 1.72 | 4 | 0.43 | 0.98 | 0.426 |
|  | Choline | 0.24 | 4 | 0.06 | 1.52 | 0.206 |
|  | **NAA** | **9.84** | **4** | **2.46** | **4.67** | **0.002** |
|  | Creatine | 1.98 | 4 | 0.50 | 2.07 | 0.095 |
| Occipital | Aspartate | 0.94 | 4 | 0.24 | 0.50 | 0.736 |
|  | Glutamine | 0.56 | 4 | 0.14 | 1.26 | 0.295 |
|  | Glutamate | 1.54 | 4 | 0.39 | 1.33 | 0.267 |
|  | Myoinositol | 0.74 | 4 | 0.18 | 0.36 | 0.838 |
|  | Asc/GSH | 0.18 | 4 | 0.05 | 0.67 | 0.614 |
|  | Glucose/Tau | 1.48 | 4 | 0.37 | 0.64 | 0.634 |
|  | Choline | 0.02 | 4 | 0.01 | 0.33 | 0.860 |
|  | **NAA** | **3.15** | **4** | **0.79** | **3.36** | **0.014** |
|  | Creatine | 0.34 | 4 | 0.09 | 0.52 | 0.719 |

ROI: region of interest/voxel.

Asc: ascorbate. GSH: glutathione. PCh: phosphocholine. GPC: glycerophosphocholine. NAA: N-acetyl-aspartate. NAAG: N-acetyl-aspartyl-glutamate, PChr: phosphocreatine. Tau: taurine.

## Table S4: ANOVA of uncorrected MRS results

| **Within Subjects Effects** | | | | | |  |
| --- | --- | --- | --- | --- | --- | --- |
| **Cases** | | **Sum of Squares** | **df** | **Mean Square** | **F** | **p** |
| ROI | | 7.776 | 2 | 3.888 | 6.827 | 0.001 |
| ROI ✻ Diagnosis | | 20.473 | 8 | 2.559 | 4.493 | 7.323e  -5 |
| Metabolite | | 9816.48 | 8 | 1227.06 | 2371.477 | 0 |
| Metabolite ✻ Diagnosis | | 116.031 | 32 | 3.626 | 7.008 | 7.187e  -25 |
| ROI ✻ Metabolite | | 162.548 | 16 | 10.159 | 57.118 | 1.082e -131 |
| ROI ✻ Metabolite ✻ Diagnosis | | 43.263 | 64 | 0.676 | 3.801 | 7.061e  -20 |
| Diagnosis | | 43.654 | 4 | 10.914 | 5.434 | 7.411e -4 |
| **Simple Main Effects - Diagnosis** | | | | | | |
| **Level of ROI** | **Level of Metabolite** | **Sum of Squares** | **df** | **Mean Square** | **F** | **p** |
| Frontal | Aspartate | 4.574 | 4 | 1.144 | 6.521 | 1.674e  -4 |
|  | Glutamine | 1.623 | 4 | 0.406 | 2.075 | 0.094 |
|  | Glutamate | 34.518 | 4 | 8.629 | 16.824 | 1.243e  -9 |
|  | Myoinositol | 4.702 | 4 | 1.176 | 1.968 | 0.109 |
|  | Asc/GSH | 0.264 | 4 | 0.066 | 0.837 | 0.506 |
|  | Glucose/Tau | 7.353 | 4 | 1.838 | 4.605 | 0.002 |
|  | Choline | 0.262 | 4 | 0.065 | 1.274 | 0.289 |
|  | NAA | 54.463 | 4 | 13.616 | 18.494 | 2.525e -10 |
|  | Creatine | 5.695 | 4 | 1.424 | 5.422 | 7.538e  -4 |
| Temporal | Aspartate | 4.085 | 4 | 1.021 | 1.002 | 0.413 |
|  | Glutamine | 3.418 | 4 | 0.855 | 2.854 | 0.03 |
|  | Glutamate | 29.714 | 4 | 7.428 | 8.349 | 1.537e  -5 |
|  | Myoinositol | 2.619 | 4 | 0.655 | 1.515 | 0.208 |
|  | Asc/GSH | 0.675 | 4 | 0.169 | 0.748 | 0.562 |
|  | Glucose/Tau | 5.69 | 4 | 1.423 | 3.373 | 0.014 |
|  | Choline | 0.452 | 4 | 0.113 | 2.508 | 0.05 |
|  | NAA | 42.051 | 4 | 10.513 | 13.436 | 4.023e  -8 |
|  | Creatine | 8 | 4 | 2 | 5.948 | 3.643e  -4 |
| Occipital | Aspartate | 1.15 | 4 | 0.287 | 0.619 | 0.65 |
|  | Glutamine | 0.526 | 4 | 0.131 | 1.137 | 0.346 |
|  | Glutamate | 2.192 | 4 | 0.548 | 1.714 | 0.157 |
|  | Myoinositol | 0.687 | 4 | 0.172 | 0.305 | 0.874 |
|  | Asc/GSH | 0.143 | 4 | 0.036 | 0.503 | 0.733 |
|  | Glucose/Tau | 1.57 | 4 | 0.392 | 0.641 | 0.635 |
|  | Choline | 0.037 | 4 | 0.009 | 0.476 | 0.753 |
|  | NAA | 6.658 | 4 | 1.664 | 5.63 | 5.644e  -4 |
|  | Creatine | 0.301 | 4 | 0.075 | 0.359 | 0.837 |

## Figure S2: Correlation matrix of partial volume correction methods


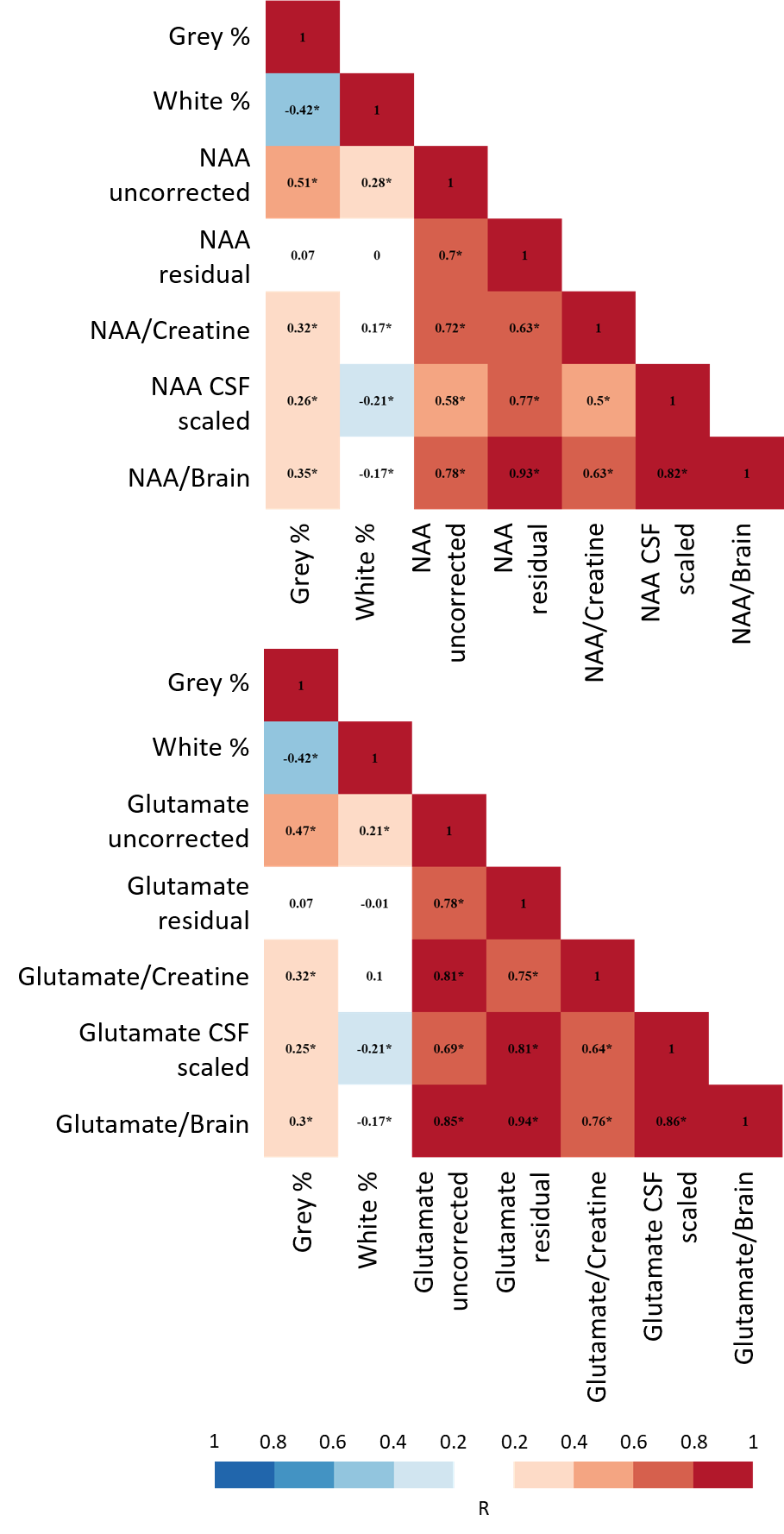
Each cell contains the regression coefficient (R) *=p<0.05 FWE corrected.
